# Effects of Agitation Speed and Kinetic Studies on Probiotication of Pomegranate Juice with *Lactobacillus casei*

**DOI:** 10.3390/molecules24132357

**Published:** 2019-06-26

**Authors:** Siti Marhaida Mustafa, Lee Suan Chua, Hesham Ali El-Enshasy

**Affiliations:** 1 Institute of Bioproduct Development, Universiti Teknologi Malaysia, Skudai 81310, Johor Bahru, Johor, Malaysia; 2 Department of Bioprocess and Polymer Engineering, School of Chemical and Energy Engineering, Faculty of Engineering, Universiti Teknologi Malaysia, Skudai 81310, Johor Bahru, Johor, Malaysia

**Keywords:** *Lactobacillus casei*, *Punica granatum*, probiotication, agitation, quercetin-3-glucoside, pomegranate

## Abstract

The issues of lactose intolerance and vegetarianism have encouraged the introduction of non-dairy fermented food into the market. Therefore, this study aims to evaluate the effect of agitation speed on the bioactive compounds and functional characteristics of probioticated pomegranate juice. Pomegranate juice was fermented with *Lactobacillus casei* at different agitation speeds ranging from 0 (microaerophilic) to 150 rpm at 37 °C. The functional properties of probioticated pomegranate juice were evaluated in terms of growth (biomass), lactic acid production, antioxidant activity, total phenolic content, and key metabolites using LC-MS/MS. The growth kinetics of fermentation was monitored at the optimal condition using one factor at a time method. High cell growth (3.58 × 10^10^ cfu/mL or 7.9 gL^−1^) was observed for *L. casei* probioticated pomegranate juice agitated at 0 rpm. The findings of this study reveal the potential of pomegranate juice as a medium for *L. casei* cultivation without nutrient supplementation. The improvement of antioxidant activity in the probioticated juice could be due to the increment of quercetin-3-glucoside. Therefore, *L. casei* grew well in pomegranate juice with a high cell viability and antioxidant activity at a non-agitated condition. Probioticated pomegranate juice is a potentially functional drink.

## 1. Introduction

Fruits are nutrient-dense foods and rich in phytonutrients with a remarkable antioxidant property for health benefits. Pomegranate (*Punica granatum* L.) is one of the widely investigated fruits in recent years, mainly due to its phytochemicals, potential uses, and health-promoting effects [1]. This could be due to its wide application in folk medicine to treat ailments, most notably type 2 diabetes, cardiovascular disease, and cancer [2]. Products derived from pomegranate usually exhibit a broad spectrum of bioactivities. This is mainly attributed to the phytochemical profile of the fruit [3]. Recent studies also prove that natural methods such as fermentation preserve the fruit quality with enhanced bioactivity [4]. The high acceptability of pomegranate juice as a cultivation medium is partly due to the awareness of the public of the adverse effects of processed foods with artificial preservatives and excessive sugar on human health.

To the best of our knowledge, fruit juices such as pear [5], watermelon [6], cantaloupe [7], orange [8], and pomegranate [9] have been fermented into non-dairy probiotic drinks using selected probiotic lactic acid bacteria [10]. Fermentation is used to deliver fruit based products without preservatives, of particularly high quality, and nutritionally healthy for consumption [11]. Fermentation is well known for its effectiveness in biopreservation, improving sensory attributes in terms of taste, aroma, and texture [12]. Biochemical reactions that take place during fermentation can also enhance the bioavailability and digestibility of phytochemicals in the human body [12]. Possibly, the process may also create new compounds that exhibit many beneficial properties for human health.

Fermentation is usually performed by lactic acid bacteria. However, their capability to adapt in the fruit ecosystem varies markedly within species and strains [13]. The metabolic response of the lactic acid bacteria affects their survival rate, which is reflected from the sensory, nutritional, and functional features of fermented foods [14]. In addition, the metabolic response of lactic acid bacteria in fruit ecosystems is also influenced by fermentation conditions such as pH, temperature, and agitation speed. Indeed, raw fruits possess intrinsic chemical and physical characteristics that provide a hostile environment for the growth of lactic acid bacteria [13]. The hostile environment could be the extreme acidic environment, buffering capacity, high concentration of carbohydrates, indigestible nutrients (fiber, inulin, and fructo-oligosaccharides), and inhibitory factors (tannins and polyphenols) [15]. Therefore, this study focuses on the effects of agitation speed and growth kinetics on the quality of probioticated pomegranate juice with *Lactobacillus casei.* Pomegranate juice probioticated with *L. casei* is believed to be a nutritious alternative with enhanced functionality.

## 2. Results and Discussion

### 2.1. Agitation Speed on the Growth of Lactobacillus casei

**Figure 1:**
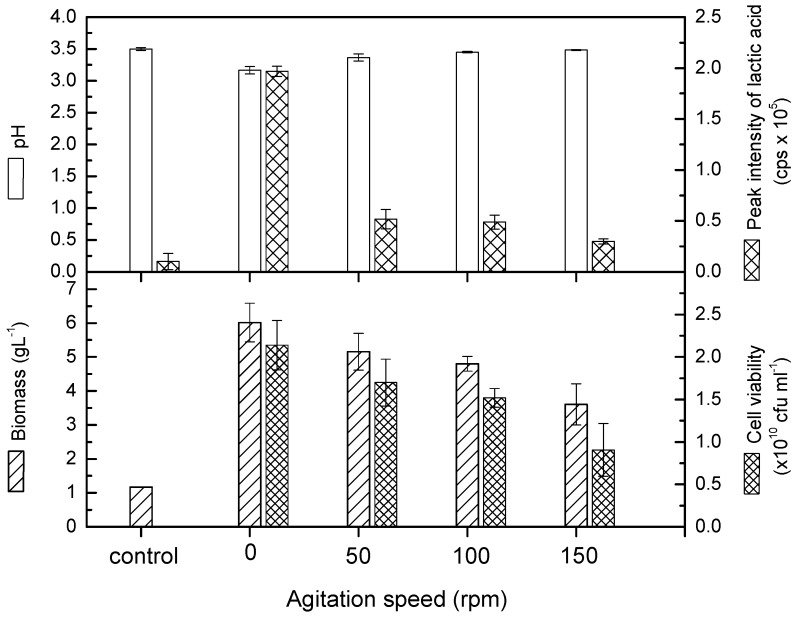
Cell viability, pH, and lactic acid content of probioticated pomegranate juice with *L. casei* at different agitation speeds. Means that do not share similar letters are significantly different.

The growth of *L. casei* in pomegranate juice could be evaluated on the basis of the biomass concentration, cell viability, and lactic acid content. The results showed that *L. casei* exhibited the highest growth in pomegranate juice at 0 rpm (Figure 1). The cell growth achieved 6.02 g/L at 0 rpm. Based on the literature, the *Lactobacillus* species favor a microaerophilic condition or require low oxygen for growth [9]. Therefore, a slow agitation rate could produce mild aeration for the species. However, the present study reveals that *L. casei* could grow well in pomegranate juice at 0 rpm and that agitation appeared to hinder the growth. The decreasing growth was noticed in terms of biomass concentration, cell viability, and lactic acid content when the agitation speed was increased from 0 to 150 rpm. In particular, the lactic acid content dropped significantly even with a mild agitation speed at 50 rpm. The reduction of lactic acid can also be seen from the slight increment of pH in the Figure 1. The initial pH of pomegranate juice increased from 3.58 to 3.17, 3.37, 3.45, and 3.49 at 0, 50, 100, and 150 rpm, respectively. This condition could be ascribed to the change of the metabolic pathway from anaerobic to aerobic conditions [16]. Zhou et al. [17] mentioned that agitation plays an important mixing and shearing role in fermentation processes. It not only improves mass and oxygen transfer between different phases, but also maintains homogeneous chemical and physical conditions in the medium. On the other hand, agitation can also cause shear forces, which influence microorganisms in several ways, such as in changes in morphology, variation in growth and metabolite formation, and can even cause damage to cell structures.

### 2.2. Agitation Speed on Total Phenolic Content

**Figure 2:**
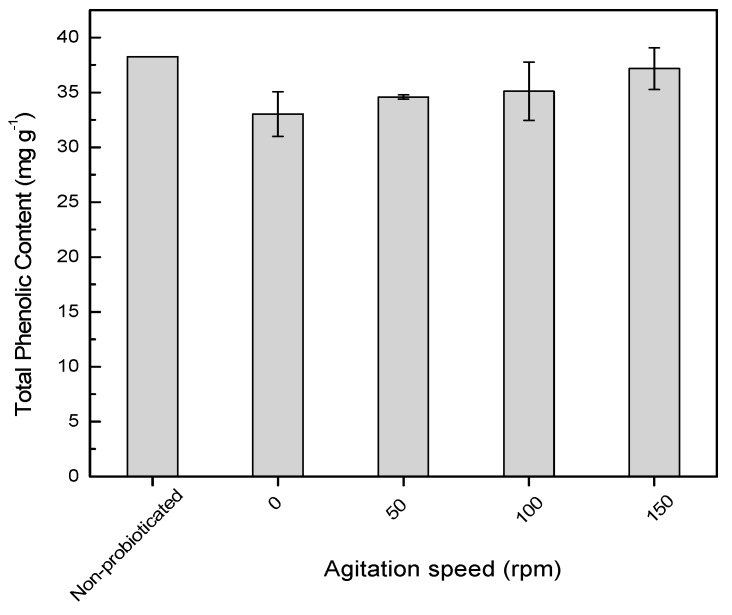
Phenolic content of non-probioticated and probioticated pomegranate juice for 24 hours at different agitation speeds. Means of total phenolic content at different agitation speeds are not significantly different.

Figure 2 shows the total phenolic content of probioticated pomegranate juice at different agitation speeds (0–150 rpm). All the agitated cultures exhibited an insignificant reduction in the total phenolic content (*p* > 0.05) compared to the samples before probiotication. The probiotication of pomegranate juice at 37 °C for 24 hours did not see any degradation of phenolic compounds in the pomegranate juice. Previous studies report that glycosylated phenolic compounds could be metabolized by the starter culture [18]. For instance, *Bifidobacteria* and *Lactobacilli* exhibit ß-glucosidase activity and participate in the hydrolysis of plant ß-glycosides [19]. Hence, the reduction of glycosylated anthocyanins results in the increment of their aglycone (anthocyanidin). The sugar moiety could be metabolized and consumed as a carbohydrate source by the selected probiotic lactic acid bacteria, and thus increase the content of aglycone after fermentation. Therefore, an agitation speed up to 150 rpm might promote hydrolysis of glyosidic bonds, but does not degrade the phenolic aglycone which was detected based on total phenolic assay.

### 2.3. Agitation Speed on Antiradical Activity

**Figure 3:**
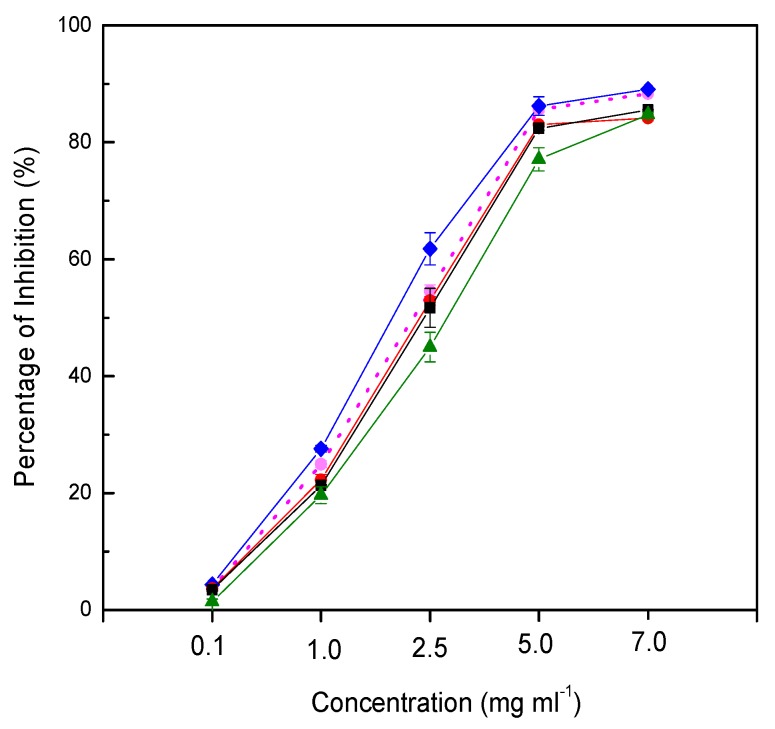
Free radicals by probioticated pomegranate juices at different agitation speeds (non-probioticated juice, dot line; 0 rpm, blue line; 50 rpm, red line; 100 rpm, black line; 150 rpm, green line).

The capacity to inhibit free radicals generated from DPPH (2,2-diphenyl-1-picrylhydrazyl) is commonly used to determine the antioxidant activity of food samples. The scavenging activity of the juice could be seen from the color change of purple to light yellow, and the change could be measured based on the absorbance spectrophotometrically. In the present study, probioticated pomegranate juice was determined for its free radical scavenging activity compared to non-probioticated juice. Pomegranate juice probioticated with *L. casei* was likely to enhance the antioxidant activity of the juice (Figure 3). However, agitation even as low as 50 rpm appeared to degrade the antiradical property of the probioticated juice. High agitation speed (150 rpm) reduced the antiradical activity significantly. Although the agitation speed did not affect the total phenolic content, the antioxidant activity of probioticated juice was significantly reduced in this study. The observation explains other metabolites. Furthermore, phenolic compounds might have partly contributed to the antiradical activity of the probioticated pomegranate juice. Another explanation is that glycosylated phenolic compounds have higher antiradical activity than their aglycones.

### 2.4. Agitation Speed on Targeted Phytochemicals

**Figure 4:**
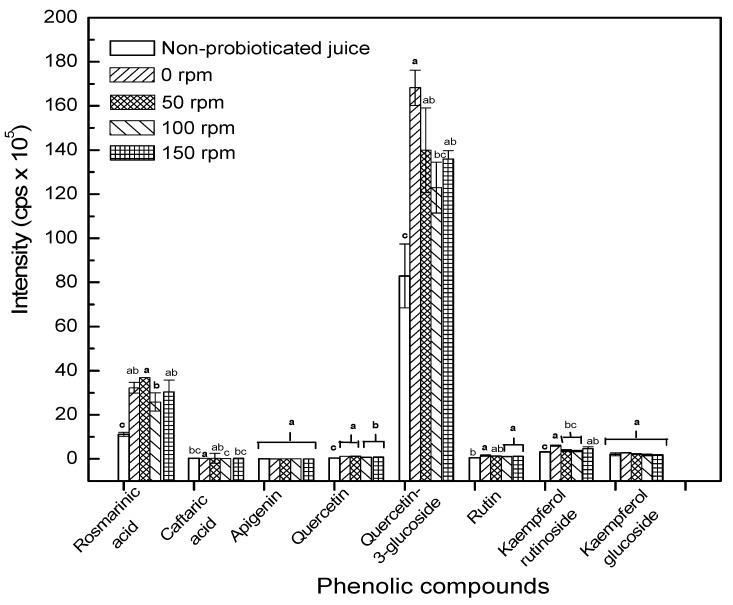
Intensity of target phenolic acids and flavonoids before and after 24-hour cultivation at different agitation speeds. Different letters of each compound indicate the significant different at *p* < 0.05.

The metabolite profile of *L. casei* probioticated pomegranate juice was compared at different agitation speeds (0–150 rpm). An optimum agitation speed could promote the microbial growth by increasing phytochemical content. Figure 4 shows that few targeted phytochemicals are significantly increased after fermentation for 24 hours. Agitation could accelerate the transfer of nutrients from liquid medium to cells for cell growth. However, agitation did not improve the metabolites content at different agitation speeds. Agitation was found to significantly decrease quercetin-3-glucoside as shown in Figure 4. The decrease might also explain the increase of its aglycone, quercetin, in this study. This also explains the decrease of antiradical activity of probioticated pomegranate juice agitated at 50–150 rpm. Csepregi et al. [20] reported that glycosylated flavonoids have higher antioxidant activity than their aglycones. The result was concomitant with the findings of Zheng et al. [21], who reported the decrease of glycosyl flavonoids from 40 to 200 rpm in the wheat germ fermentation, most probably attributed to the deglycosylation process. Furthermore, *S. cerevisiae* which is also capable of deconjugating flavonoid aglycones, might consequently reduce the concentration of flavonoid glycosides. Hence, the increase of the agitation speed improved the metabolism of *S. cerevisiae*, and thus led to a decline in the flavonoid content resulted from deconjugation [21].

Jamal et al. [22] reported that the formation of phenolic acids indicate the action of various enzymes, especially esterases and cell-wall degrading enzymes secreted by microorganisms. The agitation speed was found to affect many enzymes activities of bacteria and fungi, as well as microalgae during cultivation [23]. Rosmarinic acid (*m/z* 359/179) showed a higher intensity than caftaric acid (*m/z* 311/149) in pomegranate juice. Rosmarinic acid was significantly increased (*p* < 0.05) after 24 hours of fermentation at 0–150 rpm. The other compounds, such as caftaric acid, apigenin, and quercetin were relatively less affected by probiotication.

### 2.5. Kinetic Studies on the Growth of Lactobacillus casei

**Figure 5:**
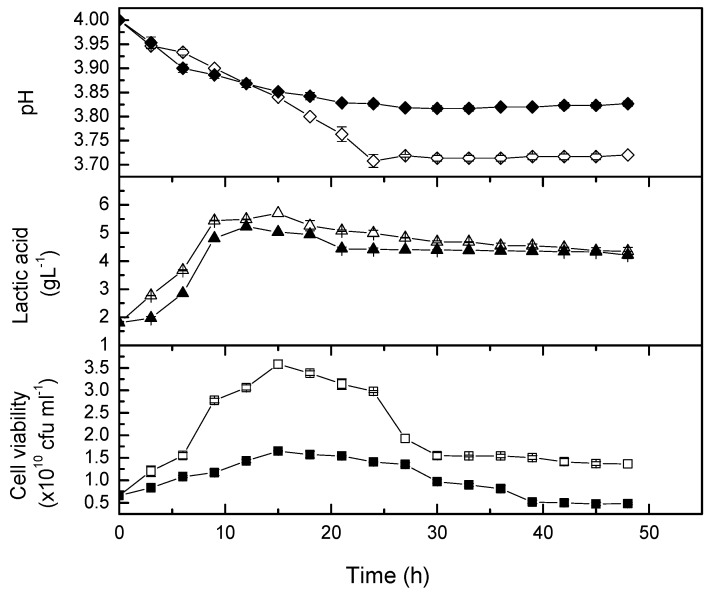
Time course of cell viability and lactic acid concentration, as well as pH changes during probiotication for 48 hours. The open symbol is growth kinetic at 0 rpm, and the closed symbol is growth kinetic at 50 rpm.

The kinetic studies on the growth of *L. casei* in pomegranate juice were investigated at static (0 rpm) and mild agitation speed (50 rpm) in shake flasks. Figure 5 illustrates that the growth rate of *L. casei* in pomegranate juice at 50 rpm was slower than that in non-agitated flasks. Even though a lower cell viability was observed, the production rate of lactic acid was marginally higher for the first 9 hours of cultivation at 50 rpm. The figure also shows that *L. casei* reached the optimum growth approximately after 15 hours of cultivation. Previously, Mousavi *et al.* [9] utilized four strains of lactic acid bacteria (*L. paracasei, L. acidophilus, L. delbruekii*, and *L. plantarum*) and recorded a minor decrease in the microbial growth at the first 24 hours of fermentation. Therefore, pomegranate juice was likely a good medium for *L. casei* because there was a short lag phase upon inoculation. According to Schultz and Kishony [24], the poor inoculum condition or low concentration of growth factors in the medium may also cause a long lag phase.

In the present study, *L. casei* was able to produce 5.7 g/L and 5.0 g/L lactic acid after being probioticated with whole fruit, squeezed pomegranate juice at 37 °C in non-agitated and 50 rpm flasks, respectively. The results are comparable with previous findings. Mousavi et al. [9] reported a significant increase of lactic acid concentration (6.1 g/L lactic acid) in pomegranate juice during the log phase of *L*. *plantarum*. The value was significantly higher than those produced by *L. acidophilus* (4.9 g/L), *L. paracasei* (4.46 g/L) and *L. delbruekii* (5.3 g/L). Around 4.6 to 5.9 g/L of lactic acid was also produced by kefir grains inoculum in a mixture of pomegranate juice and whey in the study of Sabokbar and Khodaiyan [25]. The decrease of lactic acid production in high agitation speed medium also reported by Gupta et al. [16] who fermented seaweed using *L. digitate.* The pH of the fermenting broth dropped from 5.0 to 3.5 throughout the exponential growth phase [16]. They recorded the lactic acid content, 1.25 and 1.18 g/L at 50 and 100 rpm, respectively, which was significantly less (*p* < 0.05) than that produced at 0 rpm (2.5 g/L). Hence, a static or microaerophillic condition was found to be suitable for lactic acid bacteria in seaweed fermentation as well.

The sugar profile of *L. casei* probioticated pomegranate juice was also monitored during fermentation. Glucose and fructose are dominant sugars in pomegranate juice [26]. Previously, the monosaccharides were analyzed from ten cultivars grown in Turkey [27]. The highest glucose (84.18 g/L) and fructose (83.34 g/L) content were found in the ZZ variety, while the lowest glucose (70.96 g/L) and fructose (71.23 g/L) content were recorded in the I1264 variety. The present study recorded the glucose and fructose content around 53 g/L and 81 g/L, respectively. The glucose content of pomegranate juice was slightly lower than the pomegranate cultivars from Turkey.

**Figure 6:**
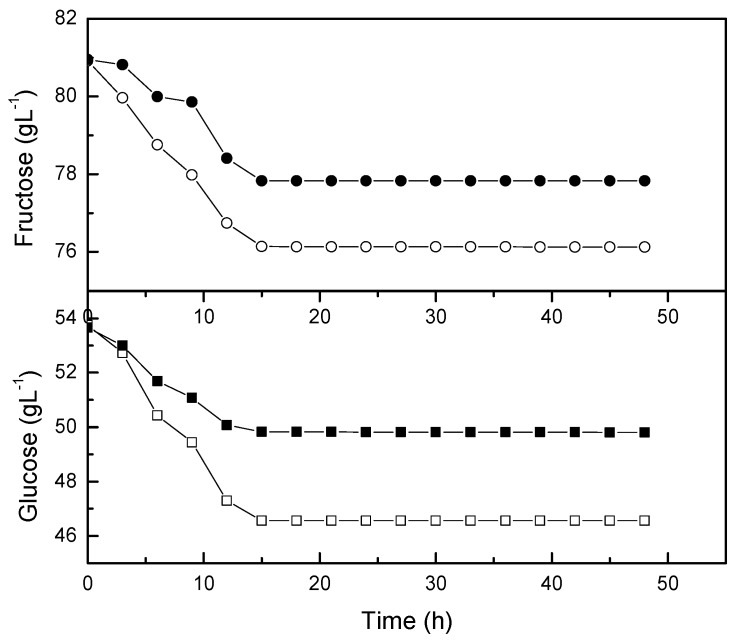
Consumption by *L. casei* in shake flasks. The open symbol is growth kinetic at 0 rpm and the closed symbol is growth kinetic at 50 rpm.

The time course of glucose and fructose reached an equilibrium after 15 hours of probiotication with *L. casei* (Figure 6). In line with this observation, the biomass and lactic acid concentration was reduced after the sugar consumption reached an equilibrium (Figure 5). The consumption rate of glucose and fructose by *L. casei* was about 10–20% higher than those rates in non-agitated flasks. The concentration of glucose was reduced more significantly in comparison with fructose during the exponential phase. Mousavi et al. [18] reported that glucose was a good carbon and energy source for *Lactobacilli* and *Bifidobacteria*. Somehow, the metabolism of carbohydrates by *Lactobacillus* varied from strain to strain in different substrates and fermentation durations [13]. Nutrients, particularly nitrogen source and minerals are necessary for lactic acid fermentation [28]. Therefore, pomegranate juice is rich in nutrients for the growth of *L. casei* for lactic acid production.

Supposedly, the agitation speed would enhance fluid-to-particle mass transfer. However, there was 70.2% reduction in the growth rate of cells as the agitation speed was increased from 0 to 50 rpm. Zotta et al. [29] revealed that the difference in growth rate was attributed to the difference in metabolic pathways for aerobic and anaerobic conditions. Oxygen inhibition associated with superoxide could be responsible for the lower cell growth rate under aerobic conditions [16].

### 2.6. Kinetics of Quercetin-3-glucoside and its Antioxidant Capacity

**Figure 7:**
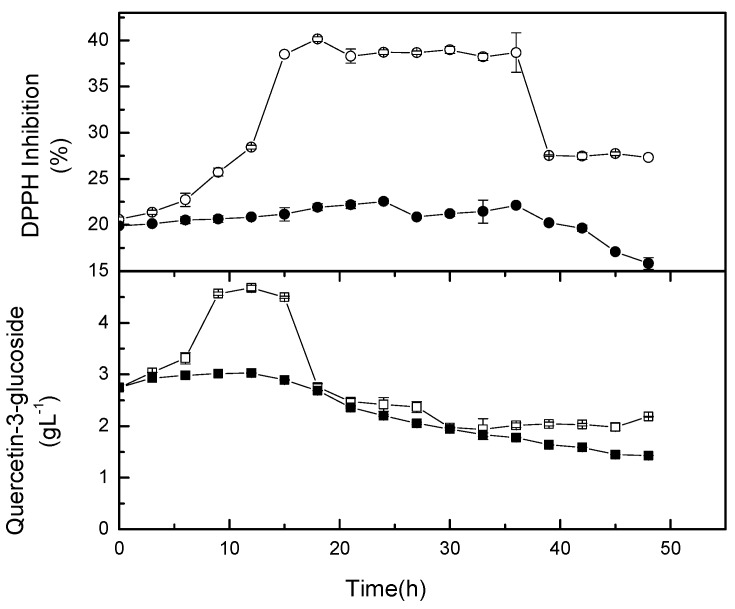
Quercetin-3-glucoside and antioxidant activity in shake flask cultures. The open symbol is growth kinetic at 0 rpm and the closed symbol is growth kinetic at 50 rpm.

Quercetin-3-glucoside was identified as the most intense compound, and therefore, its kinetic was monitored during fermentation. The increase of the glycosylated quercetin could be due to glycosylation (Figure 7). Glycosylation happened for the first 15 hours, and was then followed by deglycosylation in the non-agitated flasks. However, no significant increment of quercetin-3-glucoside was observed for broth agitated at 50 rpm. Mild agitation might speed up the hydrolysis of β-glucosidase to break down compounds containing β-glucosidic linkage [30]. Huang et al. [31] reported that the conversion of rutin to quercetin-3’-monoglucoside, and subsequently quercetin (aglycone) could be attributed to the β-glucosidase activity either from *Toona sinensis* leaves or the dominant lactic acid bacteria, namely *L. plantarum* or *Leuconostoc mensenteroides* in the fermented silage. In another study, Tranchimand et al. [32] reported that rutin can be hydrolysed by α-rhamnosidase to produce quercetin-3-glucoside, and then hydrolysed further by β-glucosidase to produce quercetin. Cho et al. [33] showed that the increase of flavonols in fermented soybean by *Bacillus pumilus* HY1. Flavonol glycosides were reduced during fermentation due to oxidative degradation. This was due to the esterase and tannase activities of lactic acid bacteria during fermentation. The findings suggest that some metabolites could be degraded during fermentation and/or new metabolites may be produced from the degraded compounds. Rodriguez et al. [34] reported the degradation of tannins and phenolic acids by lactic acid bacteria, specifically *L. plantarum* in foods. The degradation was due to the activity of tannase and phenolic acid decarboxylase from the bacteria. Therefore, the degraded tannins and phenolic compounds are possibly converted to antioxidative compounds [34]. On the other hand, Baghbadorani et al. [35] reported that the increase in the antioxidant activity of fermented soymilk could be due to the liberation of isoflavone through the catalytic action of β-glucosidase and intracellular antioxidants of starter organisms. The increase or decrease of the antioxidant activity is dependant upon either a factor or factors in synergistic effect [5].

Most studies agree that the fermentation of food using lactic acid bacteria could improve its antioxidant activity. As reported by Kachouri et al. [36], the antioxidant activity was increased in *L. plantarum* LAB 1 fermented olive culture. The increase is most probably due to the production of metabolites that have high antioxidant capacity to scavenge free radicals. Figure 7 also shows that the metabolites in non-agitated flasks have a higher antiradical property than those metabolites in mild agitation culture. The observation explains that agitation may lead to different mechanistic pathways of *L. casei* during fermentation.

## 3. Materials and Methods

### 3.1. Pomegranate and Chemicals

The fruits of pomegranate (*Punica granatum* cv. Indian red “Ruby”) was sourced from Ecotech Univarsal Pvt Ltd (Mumbai, Maharashtra, India). Only ripe fruits in good appearance without cuts or cracks on the husk were selected as samples in this study. Hygienic preparation was practiced during sample handling and juicing. The whole piece of pomegranate was cut into smaller portions and then blended into juice. The thick husk puree was sterile filtered by cellulose acetate membrane (0.45 µm) to produce dark reddish-brown juice. The juice was then centrifuged and aliquoted before kept in a freezer (−20 °C). Lactic acid, 2,2-Diphenyl-1-picrylhydrazyl (DPPH) and Folin & Ciocalteu’s phenol reagent were purchased from Sigma-Aldrich (St. Louis, MO, USA). DeMann, Rogosa and Sharpe (MRS) agar, glucose, and fructose were bought from Merck (Darmstadt, Germany).

### 3.2. Probiotication at Different Agitation Speeds

*Lactobacillus casei* subsp. *casei* (NRRL B-1922) was inoculated in de Man, Rogosa and Sharpe (MRS) broth for 24 hours at 30 °C under microaerophilic condition according to the procedures described by Mousavi et al. [9]. Probiotication was carried out by adding 10% *w/v Lactobacillus casei* into pomegranate juice (30 mL) in a 50 mL conical flask. The fermentation was carried out for 24 hours at 37 °C with different agitation speeds ranging from 0 to 150 rpm. The effects of the agitation speed on the concentration of biomass, total phenolic content, antiradical activity, lactic acid, sugars (glucose and fructose), and other small metabolites were evaluated in this study. The effects of the agitation speed on the concentration of biomass (g/L), total phenolic content (mg/g), antiradical activity (% inhibition), lactic acid (counts per second, cps), and other small metabolites (counts per second) were evaluated in this study.

### 3.3. Determination of Biomass Cconcentration

The biomass concentration was used to express cell growth which was determined by measuring the optical density of cell suspension at 590 nm. The difference between the initial and final absorbance corresponds to the microbial growth during fermentation. The calibration curve of cell growth was constructed based on the concentration of biomass (g/L) against the optical density of the bacterial strain.

### 3.4. Determination of Microbial Count

The standard plate count method was used to determine the bacterial count in samples after probiotication. A serial dilution of samples was prepared and 1 mL of each diluted sample was transferred to the test tube containing 9 mL saline solution. MRS agar, which was used to enumerate bacteria in probioticated samples, was sterilized in an autoclave at 121 °C for 20 minutes before use. One milliliter of diluted sample was transferred to a sterilized petri dish and 25 mL agar was poured into the petri dish. The dish was swirled in both clock- and anti-clock wise direction for uniform mixing. The sample dish was kept in upside down position after the agar solidified and incubated at 37 °C for 24 hours. The formation of bacterial colonies was counted and expressed as cfu/mL.

### 3.5. Determination of Total Phenolic Content

The total phenolic content of pomegranate juice was evaluated based on the procedures described by Mahboubi et al. [37]. A 200 µL centrifuged pomegranate juice was mixed with 1.5 mL of 10-fold diluted Folin and Ciocalteu’s phenol reagent. Next, 1.5 mL of 7.5% sodium bicarbonate was added to the mixture and then incubated at room temperature for 60 min before measurement at 760 nm using a spectrophotometer (UV-1800, Shimadzu, Nakagyo-ku, Kyoto, Japan). The results were displayed as gallic acid equivalents (mg GAE /g sample).

### 3.6. Determination of Antiradical Activity

The antiradical activity was analyzed using free radical DPPH assay [18]. DPPH (0.1 mM) solution was prepared in methanol. A 300 µL of the solution was mixed with 2.7 mL of the probioticated pomegranate juice. Samples were incubated at room temperature in dark condition for 30 min. The absorbance was recorded at 517 nm using a spectrophotometer. The ability to scavenge the DPPH radicals was calculated as the percentage of scavenged DPPH using Equation (1).

(1) $$\%\;\mathrm{radical}\;\mathrm{scavenging}=\left[\frac{A1-A2}{A1}\right]\times \;100$$

A1 is the absorbance of blank and A2 is the absorbance of sample (pomegranate juice).

### 3.7. Kinetic Studies on Probiotication

*L. casei* was cultivated and monitored at the optimized conditions of probiotication for 48 hours in shake flasks (50 mL). The kinetic data of probiotication was recorded at 0 and 50 rpm for comparison. A sample was withdrawn for 3-hour interval to determine the time course of biomass accumulation, lactic acid concentration, pH change, sugar consumption, and target metabolite quantitation. The experiment was repeated in triplicate individually.

### 3.8. Lactic Acid and Sugar Quantification by HPLC

The concentration of lactic acid was analyzed using HPLC (Agilent 1200 Series, Santa Clara, CA, USA) according to the method proposed by Sabokbar and Khoidayan [25] and Lai et al. [38], with some modifications. Samples (2.0 mL) were filtered using 0.22 μm nylon filters before injection. The injection volume was 20 µL using an autosampler (Agilent HiP-ALS, G1367B, Santa Clara, CA, USA). Chromatographic separation was performed by using ROA-Organic Acid H+ (Phenomenex, Torrance, CA, USA) column (300 × 7.80 mm, 8 µm). The column was kept in a column compartment (Agilent TCC, G1316A, Santa Clara, CA, USA) and maintained at 40 °C. The mobile phase was 0.005 N sulphuric acid (H_2_SO_4_) with a flow rate of 0.8 mL min^−1^ in isocratic elution for 20 min. Lactic acid was detected at 210 nm using a UV detector (Agilent MWD, G1365B, Santa Clara, CA, USA), whereas sugars (glucose and fructose) were monitored using a refractive index detector (Agilent RID, G1362A, Santa Clara, CA, USA). The target compound was quantified by comparing its peak area with the standard curve constructed using standard chemicals.

### 3.9. Metabolite Identification by LC-MS/MS

The analytical UPLC, Waters Acquity (Milford, MA, USA) system was coupled with a triple quadrupole-linear ion trap tandem mass spectrometer (Applied Biosystems 4000 Q TRAP; Life Technologies Corporation, Carlsbad, CA, USA) with an electrospray ionisation (ESI) source. A C18 reserved phase Acquity column (150 × 4.6 mm, 1.7 µm) protected by a guard column was used to determine the presence of target phenolic acids and flavonoids using the negative ion mode of multiple reaction monitoring (MRM). The mobile phase was a binary solvent system consisting of solvent A (water with 0.1% formic acid) and solvent B (acetonitrile). The UPLC gradient was: Zero to five min, 10% B; 5–15 min, 10–90% B; 15–20 min, 90% B; 20–25 min, 90–10% B; 25–30 min, 10% B for final washing and equilibration of the column for the next run. The flow rate was 0.2 ml/min and the injection volume was 5 µL. All samples were filtered with 0.2 µm nylon membrane filter prior to injection.

### 3.10. Statistical Analysis

At least three replicate data were subjected to one-way analysis of variance (ANOVA), and pairwise comparison of treatment means were measured by Tukey’s procedure at a *p* value of 0.05 using the statistical software, Minitab 16 (Minitab Pty Ltd, Sydney, Australia). The function of one-way ANOVA was used to determine the differences among means of data.

## 4. Conclusions

The present study found that *L. casei* could grow well in pomegranate juice without agitation. Mild agitation at 50 rpm appeared to hindrance the growth of the strain (14.3% reduction) and degrade the antioxidative compounds (quercetin-3-glucoside). This was possibly due to the different pathways followed by *L. casei* under different agitation conditions. This study concludes that non-agitation condition is favorable for the production of *L. casei* probioticated pomegranate juice in shake flasks. Nevertheless, the optimum agitation speed may be different if the probiotication is carried out in an industrial-scale process. Mild agitation speed may be required, or alternatively, mild agitation could be integrated with mild aeration to assist mixing with minimal effects on cell structure and its growth profile. The probioticated pomegranate juice is likely to benefit consumers with a lactose intolerance problem.
